# MAGiC: A Multimodal Framework for Analysing Gaze in Dyadic Communication

**DOI:** 10.16910/jemr.11.6.2

**Published:** 2018-11-12

**Authors:** Ülkü Arslan Aydın, Sinan Kalkan, Cengiz Acartürk

**Affiliations:** Cognitive Science Program Middle East Technical University Ankara, Turkey; Computer Science Department Middle East Technical University Ankara, Turkey

**Keywords:** Gaze analysis, speech analysis, automatic face detection, automatic speech segmentation

## Abstract

The analysis of dynamic scenes has been a challenging domain in eye tracking research. This study presents a framework, named MAGiC, for analyzing gaze contact and gaze aversion in face-to-face communication. MAGiC provides an environment that is able to detect and track the conversation partner’s face automatically, overlay gaze data on top of the face video, and incorporate speech by means of speech-act annotation. Specifically, MAGiC integrates eye tracking data for gaze, audio data for speech segmentation, and video data for face tracking. MAGiC is an open source framework and its usage is demonstrated via publicly available video content and wiki pages. We explored the capabilities of MAGiC through a pilot study and showed that it facilitates the analysis of dynamic gaze data by reducing the annotation effort and the time spent for manual analysis of video data.

## Introduction

In face-to-face social communication, interlocutors exchange both verbal and non-verbal signals. Non-verbal signals are conveyed in various modalities, such as facial expressions, gestures, intonation and eye contact. Previous research has shown that non-verbal messages prevail synchronous verbal messages in case of a conflict between the two. In particular, interlocutors usually interpret non-verbal messages rather than verbal messages as a reflection of true feelings and intentions (1, 2). Therefore, an investigation of the structural underpinnings of social interaction requires the study of both non-verbal modalities and verbal modalities of communication. In the present study, we focus on gaze as a non-verbal modality in face-to-face communication. In particular, we focus on eye contact and gaze aversion.

Eye contact is a crucial signal for social communication. It plays a major role in initiating a conversation, in regulating turn taking (3, 4), in signaling topic (5, 6, 7, 8) and in adjusting the conversational roles of interlocutors (9, 10, 11). Moreover, interlocutor’s putative mental states, such as *interest*, are usually inferred from gaze (12). In particular, eye contact is a fundamental, initial step for capturing the attention of the communication partner and establishing joint attention (13, 14).

Gaze aversion is another coordinated interaction pattern that regulates conversation. Gaze aversion is the act of intentionally looking away from the interlocutor. The previous research has explored the effects of gaze aversion on avoidance and approach. These studies have shown that an averted gaze of an interlocutor initiates a tendency to avoid, whereas a direct gaze initiates a tendency to approach (15). Similarly, the participants give higher ratings for likeability and attractiveness when picture stimuli involve a face with a direct gaze contact, compared to the stimuli that involve a face with averted gaze (16, 17).

The conversational functions of gaze aversion are also closely related to speech (18, 19, 20). In particular, gaze provides repeating, complementing, regulating and substitution of a verbal message. Speech requires complementary functions, such as temporal coordination of embodied cognitive processes including planning, memory retrieval for lexical and semantic information, and phonemic construction (21, 22, 23, 24, 25).

A closer look at speech as a communication modality reveals that speech carries various useful signals about the content or quality of speech itself, such as intonation, volume, pitch variations, speed and actions done through speech (viz. speech acts). In the present study, we focus on *speech acts* due to its salient role as the speech modality in conversation. According to the speech act theory (26, 27), language is a tool to perform acts, as well as to describe things and inform interlocutors about them.

The speech act theory is concerned with the function of language in communication. It states that a speech act consists of various components that have distinct roles. For analyzing language in communication, discourse should be segmented into units that have communicative functions. The relevant communicative functions should be identified and labelled accordingly. The speech acts are usually identified by analyzing the content of speech. However, temporal properties of speech convey information to the interlocutor, too. For instance, the analysis of a pause may be conceived as a signal for a shift in topic (24) Similarly, a pause may be an indicator of speaker’s fluency (28) and even for and indicator of a speech disorder (29). The framework that we present in this study (viz. MAGiC) enables researchers to perform analyses by employing both content of speech and its temporal properties. In the following section, we present a major challenge that MAGiC proposes a solution, namely gaze data analysis in dynamical scenes.

## Gaze Data Analysis in Dynamical Scenes

Eye tracker manufactures have been providing researchers with the tools for identifying basic eye movement measures, such as gaze position and duration, as well as a set of derived measures, such as Area of Interest (AOI) statistics. The study of gaze in social interaction, however, requires more advanced tools that would enable the researcher to automatically analyze gaze data on dynamical scene recordings. The analysis of gaze data in dynamical scenes has been a well-acknowledged problem in eye tracking research (30) largely due to the technical challenges in recognizing and tracking objects in a dynamic scene. This is because eye trackers generate a raw data stream, which contains a list of points-of-regard (POR) during the course of tracking the participant’s eyes. In a stationary scene, it is relatively straightforward to specify sub-regions (i.e., Areas of Interest, AOIs) of the stimuli on the display. This specification is then used for extracting AOI-based eye movement statistics. In case of a dynamical scene (cf. mobile eye-trackers), the lack of predefined areas leads to challenges in automatic analysis of gaze data. A number of solutions have been proposed to improve dynamic gaze data and to make it more robust against human errors in manual data annotation, such as using infrared markers, employing inter-rater analysis and combining the state-of-the-art object recognition techniques for image processing. However, each method has its own limitations (31, 32, 33). For instance, infrared markers may lead to visual distraction. In addition, in case of multiple object detection, markers are not economically or ergonomically feasible since they should be attached to each individual object to be tracked as reported by the previous research (1). To the best of our knowledge, there is no commonly accepted method for achieving eye movement analysis in dynamic scenes as reported by the previous research. In this study, we propose a solution to this problem in a specific domain, i.e., dynamic analysis of face, as presented in the following section.

We focus on a relatively well-developed subdomain of object recognition: Face recognition. The recognition of faces has been subject to intense research in computer vision due to its potential and importance in daily life applications, e.g. in security. Accordingly, MAGiC employs face recognition techniques to automatically detect gaze contact and gaze aversion in dynamic scenarios, where eye movement data are recorded. It aims at the analysis of dynamic scenes by reducing the effort on time-consuming and error-prone manual annotation of gaze data.

MAGiC also provides an environment that facilitates the analysis of audio recordings. Manual segmentation of audio recordings into speech components and pause components is not efficient and reliable, since it may exclude potentially meaningful information from the analyses (36, 37). In the following section we report a technical overview of the framework by presenting its components for face tracking and speech segmentation.

## A Technical Overview of the MAGiC Framework

In the two subsections below, we present how *face tracking* and *speech segmentation* are conducted by MAGiC through its open source components.

### Face Tracking

Face tracking has been a challenging topic in computer vision. In face tracking, a face in a video-frame is detected first, and then it is tracked throughout the stream. In the present study, we employ an established face tracking toolkit called *OpenFace*, an open source tool for analyzing facial behavior (38). *OpenFace* combines out-of-the-box solutions with the state-of-the-art research to perform tasks including facial-landmark detection, head-pose estimation and action unit (AU) recognition. The MAGiC’s face tracking method is based on Baltrušaitis et al. (38), Baltrušaitis, Mahmoud, & Robinson (39) , and Baltrušaitis, Robinson, & Morency (40).

**Figure 1.**
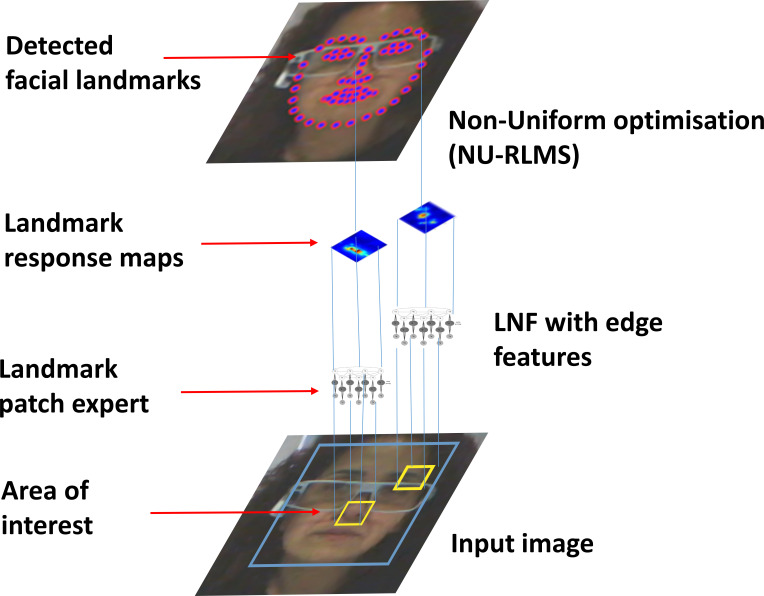
A demonstration of *OpenFace* methodology, adapted from Baltrušaitis et al. (40). It is intentionally limited to two landmarks patch expert for the sake of clarity (all photos used upon the permission of the participant).

*OpenFace* utilizes a pre-trained face detector (trained in *dlib*), which is an open source machine-learning library written in C++ (41). The Max-margin object-detection algorithm (MMOD) of the face detector uses Histogram of Oriented Gradients (HOG) feature extraction. The face detector is trained on sub-windows in an image. Since the number of windows may be large even in moderately sized images, relatively small amount of data is enough for training (41, 42). After detecting a face for detecting the facial landmarks, *OpenFace* utilizes an instance of Constrained Local Model (CLM), namely Constrained Local Neural Field (CLNF), to perform feature detection problems even in complex scenes. The response maps are extracted by using pre-trained patch experts. Patch responses are optimized with a fitting method, viz. Non-Uniform Regularized Landmark Mean-Shift (NU-RLMS, see Figure 1).

The CLM (Constrained Local Model) is composed of three main steps. First, a Point Distribution Model (PDM) extracts the mean geometry of a shape from a set of training shapes. A statistical shape model is built from a given set of samples. Each shape in the training set is characterized by a set of landmark points. The number of landmarks and the anatomical locations represented by specific landmark points should be consistent from one shape to the next. For instance, for a face shape, specific landmark points may always correspond to eyelids. In order to minimize the sum of squared distances to the mean of a set, each training shape is aligned into a common coordinate frame by rotating, translating and scaling them. The Principal Component Analysis (PCA) is used for picking out the correlations between groups of landmarks among the trained shapes. At the end of the PDM step, patches are created around each facial landmark. The patches are trained with a given set of face-shapes.

**Figure 2.**
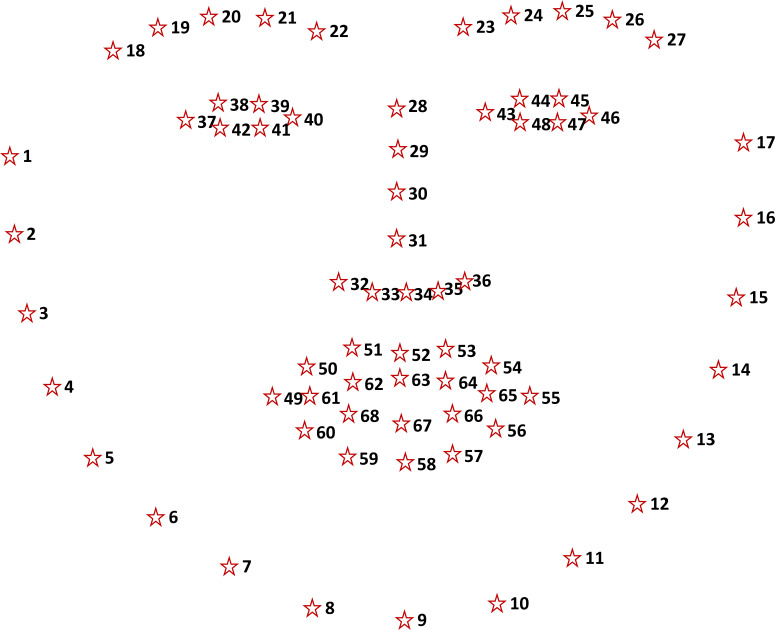
A total of 68 landmark positions on a face.

**Patch Experts**, also known as *local detectors*, are used for calculating response maps that represent the probability of a certain landmark that is being aligned at image location *x*_i_ (Eq. (1)), from Baltrušaitis et al. (40). A total of 68 patch experts are employed to localize 68 facial landmark positions, as presented in Figure 2.

**(1):**
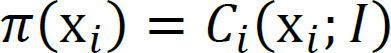


where I is an intensity image, and $C_{i}$ is a logistic regressor intercept with a value between *0* to *1* (*0* representing no alignment and *1* representing perfect alignment). Due to its computational advantages and implementational simplicity, Support Vector Regressors (SVR) are usually employed as patch experts. On the other hand, the CLNF (Constrained Local Neural Field) model uses the LNF approach, which considers spatial features that lead to fewer peaks, smoother responses and reduced noises.

**Regularised Landmark Mean Shift** (RLMS) is the next step of the CLM (Constrained Local Model). RLMS is a common method to solve the fitting problem. It updates the CLM parameters to get closer to a solution. An iterative fitting method is used to update the initial parameters of the CLM, until achieving a convergence to an optimal solution. The general concept of iterative fitting is defined in Eq. (2), adapted from Baltrušaitis et al. (40):

**(2):**
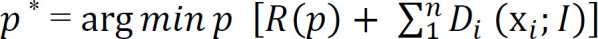


where R is a regularization term and $D_{i}$ represents the misalignment measure for image I at image location $x_{i}$. Regularizing model parameters is necessary to prevent overfitting (overfitting causes a model perform poor on data not used during training). RLMS does not discriminate between confidence levels of response maps. Due to noisy response maps, a novel non-uniform RLMS weighting mean-shifts is employed for efficiency.

At the end RLMS, the *OpenFace* toolkit detects a total of 68 facial landmarks (Figure 2). The detection of the face boundaries based on facial landmarks enables more precise calculations than using a rectangle that covers the face region.

We extended the *OpenFace* source code by making a set of improvements, which allowed the user to perform manual AOI annotation, generate visualizations that employ proposed input parameters, build a custom face detector and then use the detector to track the face, and generate separate output files depending on the input parameters. In the following section, we present the speech segmentation module.

### Speech Segmentation

Speech is a continuous audio stream with dynamically changing and usually indistinguishable parts. Speech analysis has been recognized as a challenging domain of research, since it is difficult to automatically identify clear boundaries between speech-related units. Speech analysis involves two interrelated family of methodologies, namely *speech segmentation* and *diarization*. Speech segmentation is the separation of the audio recordings into units of homogeneous parts, such as speech, silence, and laugh. Diarization is used for extracting various characteristics of signals, such as speaker identity, gender, channel type and background environment (e.g., noise, music, silence). The MAGiC framework addresses both methodologies, since both segmentation and identification are indispensable components of face-to-face conversation.

In MAGiC, we employed the *CMUSphinx* Speech Recognition System (43) by extending it for the analysis of recorded speech. *CMUSphinx* is an open source, platform-independent and speaker-independent speech recognition system. It is integrated with *LIUM*, an open source toolkit for speaker segmentation and diarization. The speech analysis process starts with feature extraction. *CMUSphinx* functions extract features, such as Mel-frequency Cepstral Coefficients (MFCC), which collectively represent power spectrum of a sound segment. It then performs speech segmentation based on Bayesian Information Criterion (44, 45).

The MAGiC framework performs two passes over the sound signal for speech segmentation. In the first pass, a distance-based segmentation process detects the *change points* by means of a likelihood measure, namely Generalized Likelihood Ratio (GLR). In the second pass, the system mixes together successive segments from the same speaker. After the segmentation, Bayesian Information Criterion (BIC) hierarchical clustering is performed with an initial set, which consists of one cluster per each segment. At each iteration, the $\Delta {\text{BIC}}_{\text{ij}}$ values for two successive clusters i and j are defined, as described by Meignier and Merlin (46), as follows:

**(3):**
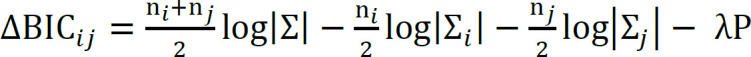


where $\left|\Sigma_{i}\right|$, $\left|\Sigma_{j}\right|$ and |Σ| are the determinants of the Gaussians associated to clusters i, j and (i+j); $n_{i}\;\text{ and }\;n_{j}\;$ refer to the total lengths of cluster i and cluster j; λ is the smoothing parameter that is chosen to get a good estimator, and P is the penalty factor. The ∆BIC values for each successive cluster are calculated and they are merged when the value is less than 0.

**Figure 3.**
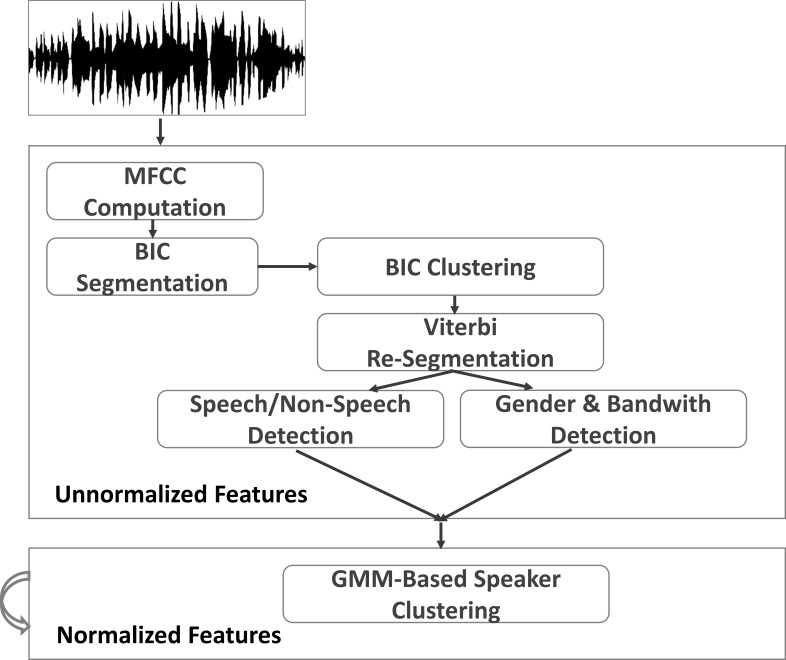
Typical workflow for speaker diarization and segmentation, adapted from LIUM Speaker Diarization Wiki Page (http://www-lium.univ-lemans.fr/diarization/doku.php/overview)

As the next step of the speech analysis, Viterbi decoding is applied for re-segmentation. A Gaussian Mixture Model (GMM) with eight components is employed to represent the clusters. The parameters of the mixture are estimated by Expectation Maximization (EM). To minimize the number of undesired segments, such as long segments or the segments that overlap with the word boundaries, the segments were slightly moved to their low energy states, and the long segments were cut iteratively to create segments that are shorter than 20 seconds. Until this stage in the workflow, non-normalized features that preserve background information are employed during segmentation and clustering. This method facilitates differentiating speakers and assigning one single speaker to each cluster. On the other hand, it may also lead to allocation of the same speaker in multiple clusters. To resolve this issue, GMM-based speaker clustering is performed with normalized features to assign the same speaker to the same cluster. The GMM iterates until it reaches a pre-defined threshold value. Figure 3 shows the workflow of speaker diarization.

We extended the *CMUSphinx* source code and made the following additions. *CMUSphinx* does not generate segments for the whole audio. For instance, it does not generate segments for the parts when the speaker could not be identified. However, those non-segmented parts might contain useful information. Thus, we carried out additional development to automatically generate audio segments of non-segmented parts. To do this, the time interval of each successive segment was calculated. If there existed a time difference between the end of the previous segment and the beginning of the next one, we created a new audio-segment that covered that time-range. We also added a new functionality for segmenting audio with specified intervals.

## Demonstration of the MAGiC Framework: A Pilot Study

This section reports a pilot study that demonstrates the functionalities and benefits of the MAGiC framework. The setting is a mock job interview setting, where a pair of participants wear eye glasses and conduct a job interview. The gaze data and the video data are then analyzed by MAGiC.

### Participants, Materials and Design

Three pairs of male participants (university students as volunteers) took part in the pilot study (mean age 28, SD = 4.60). The task was a mock job interview. One of the participants was assigned the role of an interviewer and the other an interviewee. The roles were assigned randomly. All the participants were native Turkish speakers and they had normal or corrected-to-normal vision. No time limit was introduced to the participants.

At the beginning of the session, the participants were informed about the task. Both participants wore monocular Tobii eye tracking glasses with a sampling rate of 30 Hz with a 56°x40° recording visual angle capacity for the visual scene. The glasses recorded the video of the scene camera and the sound, in addition to gaze data. The IR (infrared)-marker calibration process was repeated until reaching 80% accuracy. After the calibration, the participants were seated on the opposite sides of a table, approximately 100 cm away from each other. A beep sound was introduced to indicate the beginning of a session, for synchronization in data analysis.

Eight common job interview questions, adopted from Villani, Repetto, Cipresso, & Riva (47), were presented to an interviewer on a paper. The interviewer was instructed to ask the given questions, and also to evaluate the interviewee per each question by using paper and pencil.

### Data Analysis

We conducted data analysis using the speech analysis module, the AOI analysis module and the summary module in MAGiC. As a test environment, a PC was used with an Intel Core i5 2410M CPU at 2.30 GHz with 8 GB RAM running Windows 7 Enterprise (64 bit).

**Speech Analysis**. First, a MAGiC function (“Extract and Format Audio”) was employed to extract the audio and then to format the extracted audio for subsequent analysis. This function was run separately for each participant in the pair. Therefore, in total, six sound (.wav) files were produced. Each run took one to two seconds for the extraction. Second, the formatted audio files were segmented one by one. Audio-segments and a text file were created. The text file contained the id number and the duration of each segment. The number of segments varied depending on the length and the content of the audio (Table 1). Each run took one to two seconds for the analysis.

**Table 1.** Audio length and the number of segments for each participant’s recording.

	Interviewer/ Interviewee	
	Audio Length (m:ss.ms)	Number of Segments
Pair-1	3:46.066/ 3:57.00	170/176
Pair-2	5:25.066/ 5:40.00	120/200
Pair-3	5:28.000/ 5:09.00	246/208

Third, time-interval estimation, synchronization and re-segmentation were performed for each pair by using an interface that we call the “Time Interval Estimation” panel.

When the experiment session is conducted with multiple recording devices, one of the major issues is synchronization of the recordings. Currently, eye tracker manufacturers do not provide synchronization solutions. In most cases, the device clocks are set manually. MAGiC provides a semi-automatic method for synchronizing multiple recordings from a participant pair. In this method, the user is expected to specify the initial segment of the session in both recordings. Since, user identifies the beginning of sessions by listening to automatically created segments instead of a whole speech, this results in more accurate time estimation. Then, MAGiC calculates the time offset to provide synchronization by taking the time difference of the specified initial segments. After performing the re-segmentation process (by utilizing synchronization information and by merging segments from both recordings), we end up with equal-length session intervals for participants within each pair. The closer the microphone is to a participant, the cleaner and better the gathered audio recording is. Thus, segmentation of multiple recordings from the same session may result in different number of segments. A re-segmentation process merges segments from different recordings in order to reduce data loss. Table 2 presents the experiment duration in milliseconds and the number of segments produced after re-segmentation in our pilot study. Each run took one to two seconds.

**Table 2.** Audio length and number of segments for each participant’s recording. The number of segments increased after re-segmentation. (see Table 1)

	Exp. Duration (m:ss.ms)	Number of Segments
Pair-1	3:02.40	261
Pair-2	5:05.40	282
Pair-3	4:42.60	406

Finally, speech annotation was performed. A list of pre-defined speech acts was prepared as the first step of the analysis: Speech, Speech Pause, Thinking (e.g., “uh”, “er”, “um”, “eee”, for instance), Ask-Question, Greeting (e.g., “welcome”, “thanks for your attendance”), Confirmation (e.g., “good”, “ok”, “huh-huh”), Questionnaire Filling (Interviewer filling in questionnaire), Pre-Speech (i.e., warming up the voice), Reading and Articulation of Questions, Laugh, Signaling end of the speech (e.g., “that is all”).

The next step was the manual annotation process. For the end user, this process involved selecting the speech act(s) and annotating the segments. At each annotation, a new line was appended and displayed, which contained the relevant segment's time-interval, its associated participant (if any) and user-selected speech-act(s). AOI analysis was performed for the three pairs of participants separately. Each run took ten to twenty minutes, depending on the session-interval.

**AOI Analysis**. All six video recordings of the pilot study were processed with *OpenFace*’s *default-mode face detector*. The tracking processes produced two-dimensional landmarks on the interlocutor’s face image. The process took 4 to 10 minutes per video. Then, the gaps with at most two frames-duration were filled in by linear interpolation of raw gaze data. The raw gaze data file included the frame number, gaze point classification (either *Unclassified* or *Fixation*), and x-y coordinates. The processed data comprised 2% of total raw gaze data (Table 3). The gap filling process took less than a second per pair.

**Table 3.** The number and ratio of the filled gaps for each participant’s raw gaze data.

	Interviewer/ Interviewee	
	Number of filled gaps	Ratio of filled gaps (%)
Pair-1	146 / 236	2.15 / 3.32
Pair-2	171 / 236	1.75 / 2.31
Pair-3	157 / 335	1.60 / 3.61

After the gap filling process, we performed AOI detection by setting the parameters for eye tracker accuracy and image resolution. In the present study, the size of the captured images for face tracking was 720 × 480 pixels, while the eye tracker image-frame resolution was 640 × 480. The eye tracking glasses had a reported degree of accuracy of half a degree of visual angle. The built-in scene camera recording angles of the eye tracking glasses were 56 degrees horizontal and 40 degrees vertical. The seating distance between the participants was approximately 100 cm. Accordingly, the eye tracker accuracy was 4.84 pixels horizontal and 5.34 pixels vertical. The AOI detection took a couple of seconds. Table 4 presents the number and the ratio of image-frames that AOI detection failed due to undetected face. The results indicate that higher undetected-face rates were observed at the interviewer’s recordings. Nevertheless, face detection was performed with more than 90% success on average.

**Table 4.** The number and ratio of image-frames that face could not be detected.

	Interviewer/ Interviewee	
	Number of undetected	Ratio of undetected (%)
Pair-1	570 / 173	10.4 / 3.16
Pair-2	2113 / 488	23.1 / 5.33
Pair-3	1251 / 117	14.8 / 1.38

The absence of gaze data is another issue that leads to failure in AOI detection. Table 5 shows the ratio of undetected AOIs due to the absence of gaze data.

**Table 5.** The number and ratio of image-frames that raw gaze data were absent.

	Interviewer/ Interviewee	
	Number of undetected	Ratio of undetected (%)
Pair-1	3237 / 392	59.1 / 7.16
Pair-2	4762 / 1050	52.0 / 11.50
Pair-3	4010 / 1732	47.3 / 20.40

**Figure 4.**
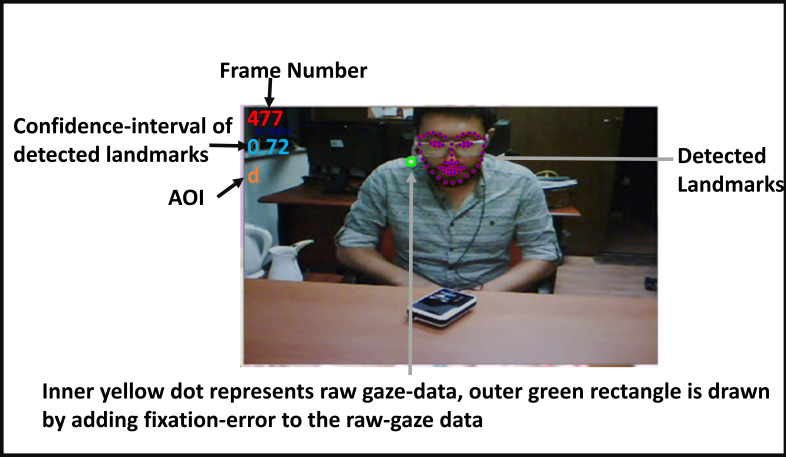
A snapshot from the visualize-tracking panel.

The failure in AOI detection on the interviewer’s side was approximately 50%. This is due to the experimental setting, where the interviewer looked at the questions to read them. This is a situation that experiment designers face frequently in dynamic experiment settings. The MAGiC framework’s interface allows the user to detect the source of the problem and to annotated it by a label through a panel interface that we name “Visualize Tracking”. The panel interface displays the recording by overlaying the detected facial landmarks, raw gaze data and gaze annotation (looking at the interlocutor’s face, i.e., in, or looking away the interlocutor’s face i.e., out) on top of the video recording for each frame, as shown in Figure 4.

The analysis of the scenes by the “Visual Tracking” panel revealed that the missing raw gaze data were due to interviewer’s reading and articulation of the questions, and evaluating the interviewee’s response by using paper and pencil. In those cases, the interviewer looked outside of the glasses frame to read the questions on the notebook. In our pilot study, the manual annotation took 15 to 20 minutes per pair, on average.

The final step in the AOI-analysis was composed of two further functions provided by the MAGiC framework: The re-analysis step merged automatically-detected AOIs with manually extracted AOI-labels. After then, the detection ratio was compared with the previous outcomes.

Table 6 shows face-detection and gaze-detection accuracies for the interviewer’s recordings. The results reveal an improvement of more than 30% after the final step, compared to the previous analysis steps (cf. Table 4 and Table 5).

**Table 6.** The number and ratio of the image-frames that face and gaze could not be detected.

	Face	
Id	Number of undetected face	Ratio of undetected face (%)
1	4	0.07
2	38	0.41
3	5	0.06
		
	Gaze	
Id	Number of undetected gaze	Ratio of undetected gaze (%)
1	1508	27.55
2	1292	14.10
3	1143	13.48

**Figure 5.**
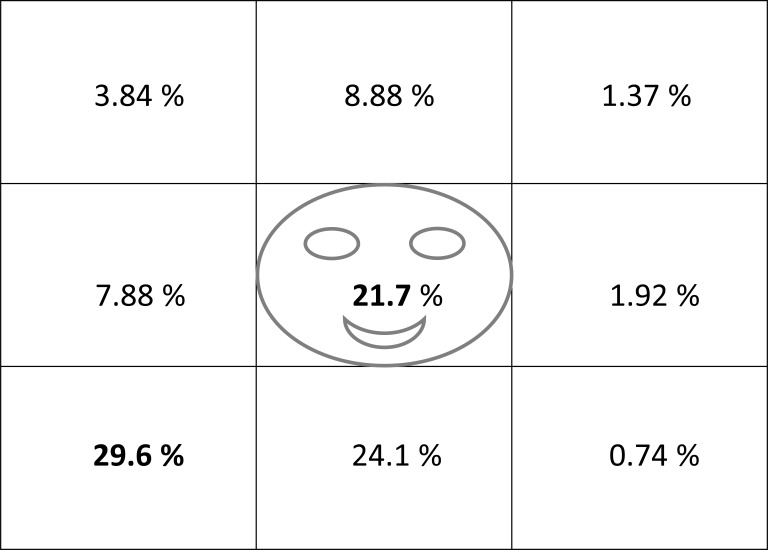
At the 21.7% of the dwell time, participants looked at interlocutor’s face. The bottom left corner with the 29.6% was the most sighted region.

The analyses also revealed the distribution of interlocutor’s gaze locations. The findings showed a tendency of more frequent gaze aversion on the right side, especially to the right-bottom (see Figure 5).

The rightward shifts are usually associated with verbal thinking, whereas leftward shifts are usually associated with visual imagery (48). On the other hand, more recent studies report that the proposed directional patterns do not consistently occur when a question elicited verbal or visuospatial thinking. Instead, the individuals are more likely to avert their gaze while a listening to a question from the partner (see Ehrlichman & Micic (49) for a review).

A further investigation of mutual gaze behavior of the conversation pairs and speech acts was conducted by a two-way ANOVA. The speech-acts had eleven levels (Speech, Speech Pause, Thinking, Ask-Question, Greeting, Confirmation, Questionnaire Filling, Pre-Speech, Reading Questions, Laugh and Signaling End of the Speech) and the mutual gaze behavior had four levels (Face Contact, Aversion, Mutual Face Contact, Mutual Aversion).

The analysis with normalized gaze distribution frequency revealed a main effect of gaze behavior, *F*(3,72) =58.3, *p*<.05. The Tukey post hoc test was performed to establish the significance of differences in frequency scores with different gaze behavior and speech-acts. It revealed that the frequency of Gaze Aversion (*M*=0.5, *SD*=0.12) was significantly larger than the frequency of Face Contact (*M*=0.1, *SD*=0.19, *p*<.05), the frequency of Mutual Face Contact (*M*=0.02, *SD*=0.06, *p*<.05), as well as the frequency of Mutual Aversion (*M*=0.38, *SD*=0.15, *p*<.05). Moreover, the frequency of Mutual Aversion was significantly larger than the frequency of Face Contact (*p*<.05) and the frequency of Mutual Face-Contact (*p*<.05), while there was no significant difference between the frequency of Face Contact and the frequency of Mutual Face Contact (*p*=0.31).

Finally, the interaction between speech-acts and gaze behavior was investigated. The results indicated that when the participants were *thinking*, there was a significant frequency difference between the frequency of Mutual Aversion (*M*=0.58, *SD*=0.07) and the frequency of Face Contact (*M*=0.03, *SD*=0.05, *p*<.05), as well as significant difference between the frequency of Mutual Aversion and the frequency of Mutual Face Contact (*M*=0.01, *SD*=0.02, *p*=.02).

## An Evaluation of the Contributions of the MAGiC Framework

In this section, we report how the MAGiC framework facilitated gaze analysis in the reported pilot study. MAGiC reduced the amount of the time spent for preparing manually annotated gaze and audio data for each image-frame of a scene video. To manually identify gaze contact and gaze aversion, and its location, a researcher would annotate 36,000 image-frames for a 10-minute session recorded by a 60 Hz eye tracker. Assuming that it takes 1 second to manually annotate a frame, the annotation would last 10 hours. MAGiC took approximately 5 to 10 minutes when it was run on a typical personal computer in today’s technology (Intel Core i5 2.3 GHz CPU and 8 GB of RAM.) The time spent for the Area of Interest (AOI) and audio annotation was also reduced. The automated annotation improved the quality of annotated data. It is difficult for human annotators to detect speech instances at this level of temporal granularity. Since full annotation is the holy grail of gaze data in dynamic analysis scenes, MAGiC also offers an interface to make manual AOI annotation to the user. This component of MAGiC is one of the pillars for improvement for future versions.

MAGiC provides the functionality for visualizing face tracking data and AOI annotation frame-by-frame. It overlays the detected facial landmarks, the raw gaze data, and the status of gaze interaction in a single video recording. It also displays the ratio of non-annotated gaze data (thus, the success level of face detection) as a percentage of total data to the user. The absence of raw gaze data or undetected faces are major reasons for the failure of an automatic AOI annotation. The user can introduce tranining to create a custom face detector for better face detection performance. The MAGiC software is licensed under the GNU General Public License (GPL). Therefore, the source code of the application is openly distributed and programmers are encouraged to study and contribute to its development. In addition to MAGiC, we also provide the modified component toolkits (OpenFace for face tracking, dlib for training of a custom face detector, and CMUSphinx for speech segmentation) on MAGiC’s github repository: MAGiC_v1.0: https://github.com/ulkursln/MAGiC/releases

## Usability Analysis of MAGiC

This section reports a usability analysis of the MAGiC framework. For the analysis, the AOI Analysis interface and the Speech Analysis interface were randomly assigned to a total of eight participants. The participants performed data analysis by using publicly available sources (see Supplementary material^1^). The usability analysis was conducted in three steps, as described below:

(1) Perform the analysis manually,

(2) Perform the analysis by using MAGiC,

(3) Asses the usability of MAGiC using 7-point scale ISO 9241/10 questionnaire.

**Figure 6.**
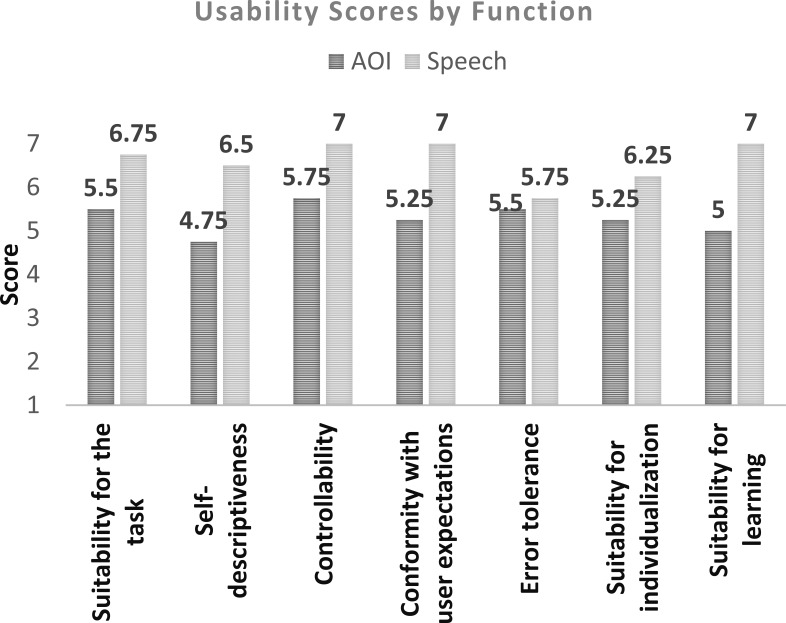
All of the usability metrics were scored higher than an average.

The Usability test scores are presented in Figure 6.

We recorded the time spent to perform data analysis, and then we compared it to the average duration when the participants performed the same analysis manually. In the AOI analysis, the mean duration to annotate a single frame decreased from 29.1 seconds (*SD*=22.7) for manual annotation to an average of 0.09 seconds (*SD*=0.02) in MAGiC. In the speech analysis, the mean duration for a single annotation decreased from 44.5 seconds (*SD*=8.8) for manual annotation to an average of 7.1 seconds (*SD*=1.4) in MAGiC.

## Discussion and Conclusion

In the present study, we introduced the MAGiC framework. It provides researchers an environment for the analysis of gaze behavior of a pair in conversation. Human-Human conversation settings are usually dynamic scenes, in which the conversation partners exhibit a set of specific gaze behavior, such as gaze contact and gaze aversion. MAGiC detects and tracks interlocutor’s face automatically in a video recording. Then it overlays gaze location data to detect gaze contact and gaze aversion behavior. It also incorporates speech data into the analysis by means of providing an interface for annotation of speech-acts.

MAGiC facilitates the analysis of dynamic eye tracking data by reducing the annotation effort and the time spent for frame-by-frame manual analysis of video data. Its capability for automated multimodal (i.e., gaze and speech-act) analysis makes MAGiC advantageous over error-prone human annotation. The MAGiC interface allows researchers to visualize face tracking process, gaze-behavior status and annotation efficiency on the same display. It also allows the user to train the face tracking components by providing labelled images manually.

The environment has been developed as an open source software tool, which is available for public use and development. MAGiC has been developed by integrating a set of open source software tools, in particular *OpenFace* for analyzing facial behavior, *dlib* for machine learning of face tracking and *CMUSphinx* for the analysis of recorded speech and extending their capabilities further for the purpose of detecting eye movement behavior, and for annotating speech data simultaneously with gaze data. MAGiC’s user interface is composed of a rich set of panels, which provide the user an environment to conduct a guided, step-by-step analysis.

MAGiC is able to process data from a single eye tracker or data in a dual eye tracking setting. We demonstrated MAGiC’s capabilities in a pilot study, which was conducted in a dual eye-tracking setting. We described MAGiC’s data analysis capabilities by describing the analysis step on the recorded data in the pilot study. We intentionally employed a low-frequency eye-tracker, with a relatively low video quality, and a low-illuminated environment, since these are typical real-environment challenges that influence face tracking capabilities. Our analysis revealed that MAGiC is able to exhibit acceptable success ratios in automatic analyses, with an average Area of Interest (AOI) labelling (i.e., gaze contact and gaze aversion detection) efficiency of approximately 80%. Likely improvements in eye tracking recording frequency, eye tracking data quality, and image resolution of video recordings have the potential to increase the accuracy of MAGiC’s outputs to better levels. We also note that MAGiC’s speech analysis component, namely *CMUSphinx* provides several high-quality acoustic models, although there is no pre-build acoustic model for Turkish. Despite this challenge, MAGiC returned successful results for the speech analysis, too. The speech-act annotation also helped us overcoming speech segmentation issues by providing sub-segments for speech.

All the data analyses were completed in approximately two hours for the three pairs of participants. Our usability analyses revealed that the time and effort spent for manual, frame-by-frame video analysis and speech segmentation takes much longer to complete, in addition to being prone to human annotator errors.

Recently, MAGiC is in its first version. Our future work will include making improvements in the existing capabilities of MAGiC, as well as developing new capabilities. For instance, face-detection ratio may be increased by employing recently published OpenFace 2.0. Also, in its current version, MAGiC sets an AOI-label on the interlocutor’s face image. We plan to expand this labelling method so that it processes other objects, such as the objects on a table. This will expand the domain of use of MAGiC into a broader range of dynamic visual environments not limited to face-to-face communication. However, this development would require training a detector for the relevant objects, which is a challenging issue for generalization of the object recognition capabilities. Moreover, the face-tracking function of MAGiC already makes it possible to extract facial expressions, based on the Facial Action Coding System (FACS). As a further improvement, MAGiC may automatically summarize facial expressions during the course of a conversation.

Finally, for speech analysis, MAGiC provides functions for semi-automatically synchronizing recordings. Further development of MAGiC may address improving its synchronization capabilities, its capability to transcribe speech into text and its capability for training speech-act annotation with pre-defined speech acts and automating subsequent annotations.

## Ethics and Conflict of Interest

The author(s) declare(s) that the contents of the article are in agreement with the ethics described in http://biblio.unibe.ch/portale/elibrary/BOP/jemr/ethics.html and that there is no conflict of interest regarding the publication of this paper.

## Footnotes

See the MAGiC App Channel under Youtube, https://www.youtube.com/channel/UC2gvq0OluwpdjVKGSGg-vaQ, and MAGiC App Wiki Page under Github
